# Publisher Correction: Single-cell transcriptomic analysis suggests two molecularly distinct subtypes of intrahepatic cholangiocarcinoma

**DOI:** 10.1038/s41467-022-30599-8

**Published:** 2022-05-17

**Authors:** Guohe Song, Yang Shi, Lu Meng, Jiaqiang Ma, Siyuan Huang, Juan Zhang, Yingcheng Wu, Jiaxin Li, Youpei Lin, Shuaixi Yang, Dongning Rao, Yifei Cheng, Jian Lin, Shuyi Ji, Yuming Liu, Shan Jiang, Xiaoliang Wang, Shu Zhang, Aiwu Ke, Xiaoying Wang, Ya Cao, Yuan Ji, Jian Zhou, Jia Fan, Xiaoming Zhang, Ruibin Xi, Qiang Gao

**Affiliations:** 1 Department of Liver Surgery and Transplantation, Liver Cancer Institute, Zhongshan Hospital, and Key Laboratory of Carcinogenesis and Cancer Invasion of Ministry of Education, Fudan University, Shanghai, China; 2 School of Mathematical Sciences and Center for Statistical Science, Peking University, Beijing, China; 3 Key Laboratory of Molecular Virology & Immunology, Institut Pasteur of Shanghai, Chinese Academy of Sciences, Shanghai, China; 4 Peking-Tsinghua Center for Life Sciences, Academy for Advanced Interdisciplinary Studies, Peking University, Beijing, China; 5 Department of Cancer Center, Jin Shan Hospital, Fudan University, Shanghai, China; 6 Department of General Surgery, Qingpu Branch of Zhongshan Hospital Affiliated to Fudan University, Shanghai, China; 7 Cancer Research Institute, Xiangya School of Medicine, Central South University, Changsha, Hunan China; 8 Department of Pathology, Zhongshan Hospital, Fudan University, Shanghai, China; 9 Key Laboratory of Medical Epigenetics and Metabolism, Institutes of Biomedical Sciences, Fudan University, Shanghai, China

**Keywords:** Cancer genomics, Tumour biomarkers, Tumour heterogeneity, Bile duct cancer

Correction to: *Nature Communications* 10.1038/s41467-022-29164-0, published online 28 March 2022.

The original version of this Article contained an error in the title, which was previously incorrectly given as ‘Single-cell transcriptomic analysis suggests two molecularly subtypes of intrahepatic cholangiocarcinoma’. The correct version states ‘Molecularly Distinct Subtypes’ in place of ‘Molecularly Subtypes’. This has been corrected in both the PDF and HTML versions of the Article.

