# Supplementary material for: Single-cell transcriptomic analysis suggests two molecularly distinct subtypes of intrahepatic cholangiocarcinoma
Source: Nat Commun. 2022 Mar 28;13:1642. doi: 10.1038/s41467-022-29164-0 (PMC8960779; doi:10.1038/s41467-022-29164-0)
Supplement: Supplementary file 1 — Supplementary Information [file 41467_2022_29164_MOESM1_ESM.pdf]

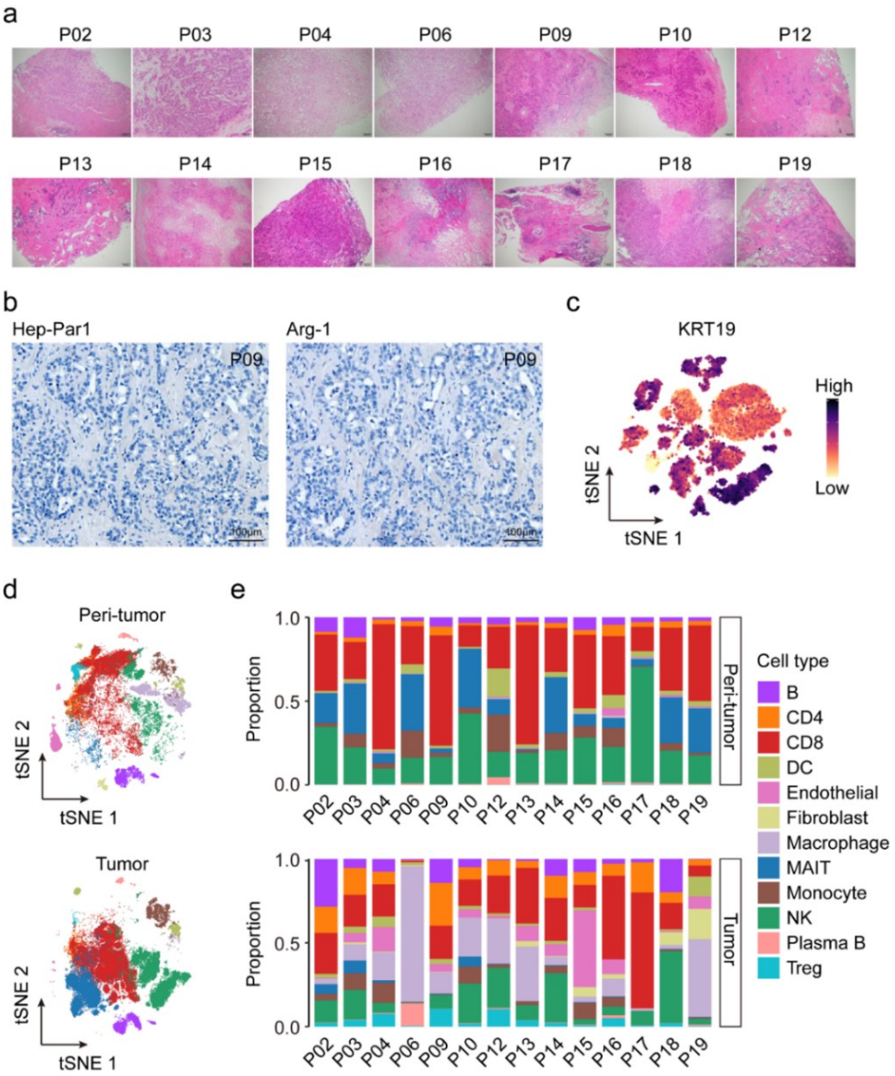

Supplementary Figure. 1 ScRNA-seq profiling of 14 iCCA. a Hematoxylin eosin (HE) staining of 14 iCCA tumors for ScRNA-seq. Experiment was repeated once with similar results. Scale bar, 100μm. **b** Representative immunohistochemistry staining revealed negative staining for Hep-Par1 and Arg-1 in P09 iCCA patient. Experiment was repeated once with similar results. Scale bar, 100μm. **c** t-SNE plot showing the expression of KRT19 in all the tumor cells. **d, e** t-SNE plots and proportions of nonmalignant cell types vary across sample origin from iCCA tumor and peritumor tissues. Source data are provided as a Source Data file.

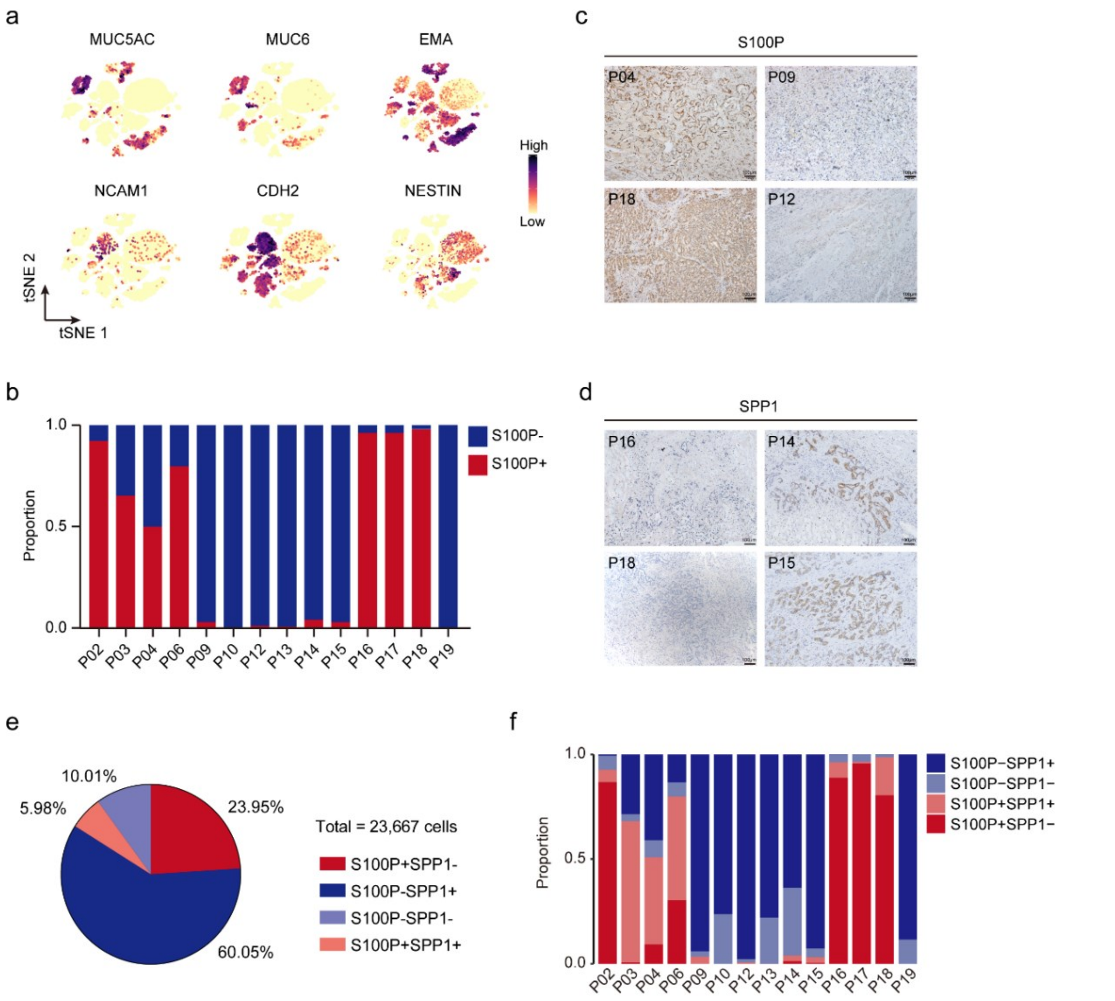

Supplementary Figure. 2 iCCA can be classified into two subtypes according to the expression of S100P and SPP1. a t-SNE plots showing the expression levels of selected marker genes in tumor cells of 14 iCCA patients. **b** Graphical representation of the proportion of S100P+ and S100P- cells in each iCCA patient. **c** Representative images of immunohistochemical expression of S100P in four scRNA-seq iCCA samples. Experiment was repeated once with similar results. Scale bar, 100μm. **d** Representative images of immunohistochemical expression of SPP1 in four scRNA-seq iCCA samples. Experiment was repeated once with similar results. Scale bar, 100μm. **e** The pie chart shows the percentage of the four groups of tumor cells in the total

tumor cells. **f** Graph of the proportions of four subtypes of iCCA cells in fourteen iCCA patients.

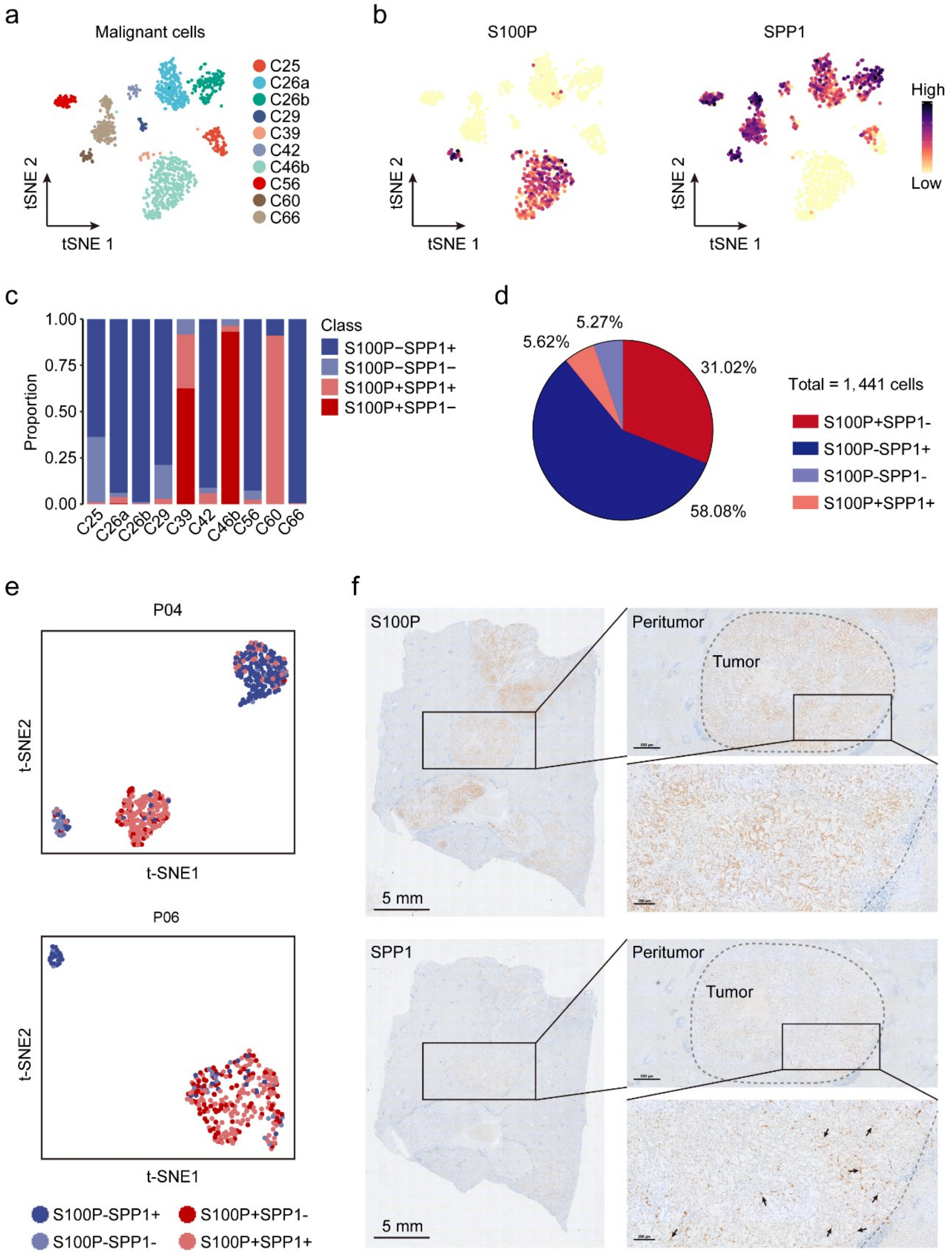

Supplementary Figure. 3 The presence of S100P+SPP1+ tumor cells in iCCA. a t-SNE plot of all malignant cells coloured by sample origin from Ma *et al.* data (Ma L, *et al.* 2019, Cancer cell). **b** t-SNE plot showing expression of

S100P and SPP1. **c** Graph of the proportions of four subtypes of iCCA cells in ten iCCA patients from Ma *et al.* data (Ma L, *et al.* 2019, Cancer cell). **d** The pie chart shows the percentage of the four groups of tumor cells in the total tumor cells from Ma *et al.* data (Ma L, *et al.* 2019, Cancer cell). **e** t-SNE plots of four subtypes of iCCA tumor cells from P04 and P06 patients from our scRNA-seq data. **f** Immunohistochemical staining for S100P and SPP1. Images were collected from 17 additional iCCA slides that contained both tumor and corresponding paracancerous tissues. Experiment was repeated once with similar results. Arrows refer to SPP1+ tumor cells. Scale bar, 5 mm (left), 200 μm (right). Source data are provided as a Source Data file.

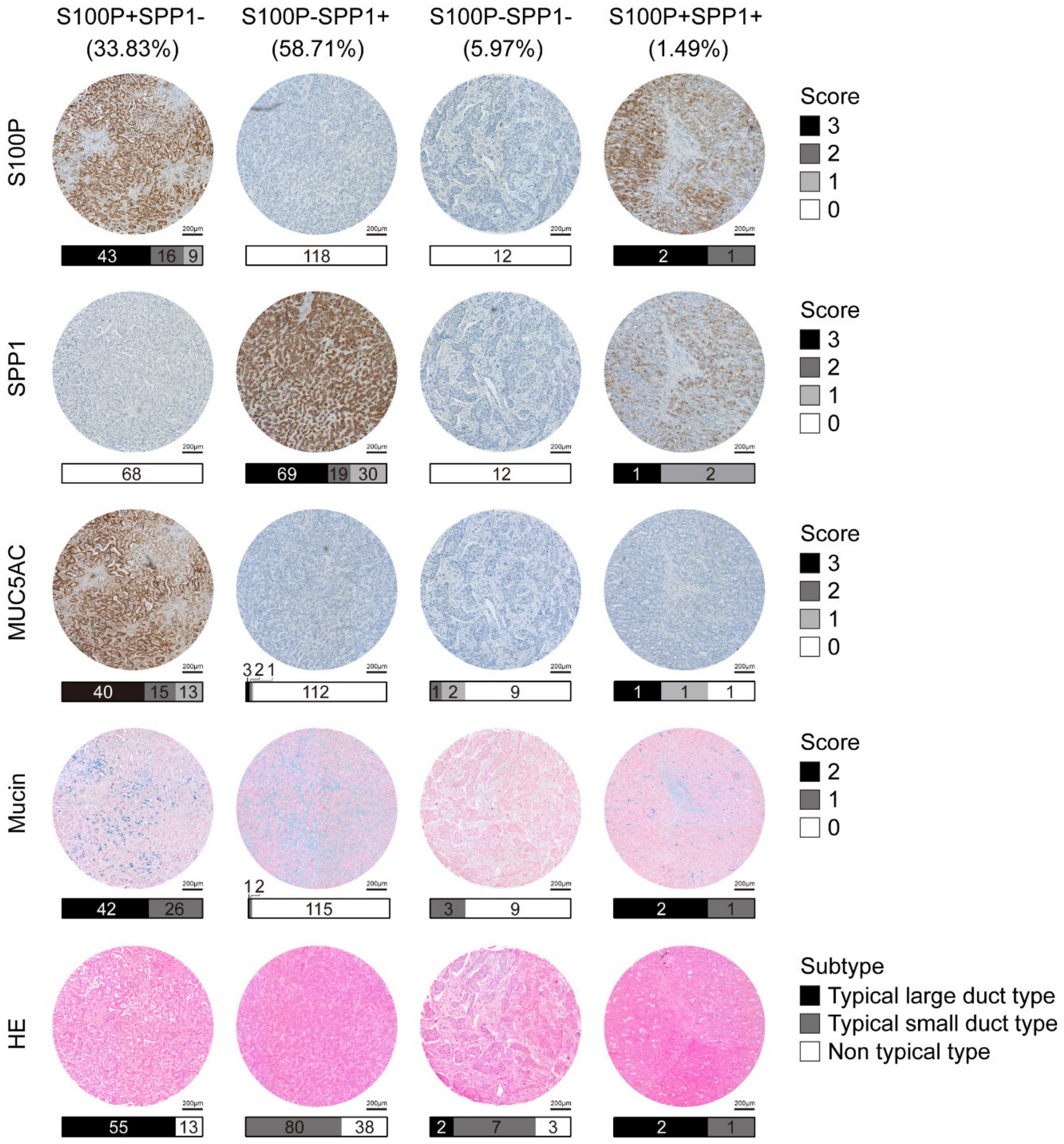

Supplementary Figure. 4 Representative immunohistochemistry staining, mucin production, and histology (hematoxylin and eosin) of 201 iCCA cases. Typical large duct type refers to iCCAs composed of columnar cells with abundant mucin production. Typical small duct type refers to iCCAs composed of cuboidal or low columnar cells with no or rare mucin production. Experiment was repeated once with similar results. Scale bar, 200 μm.

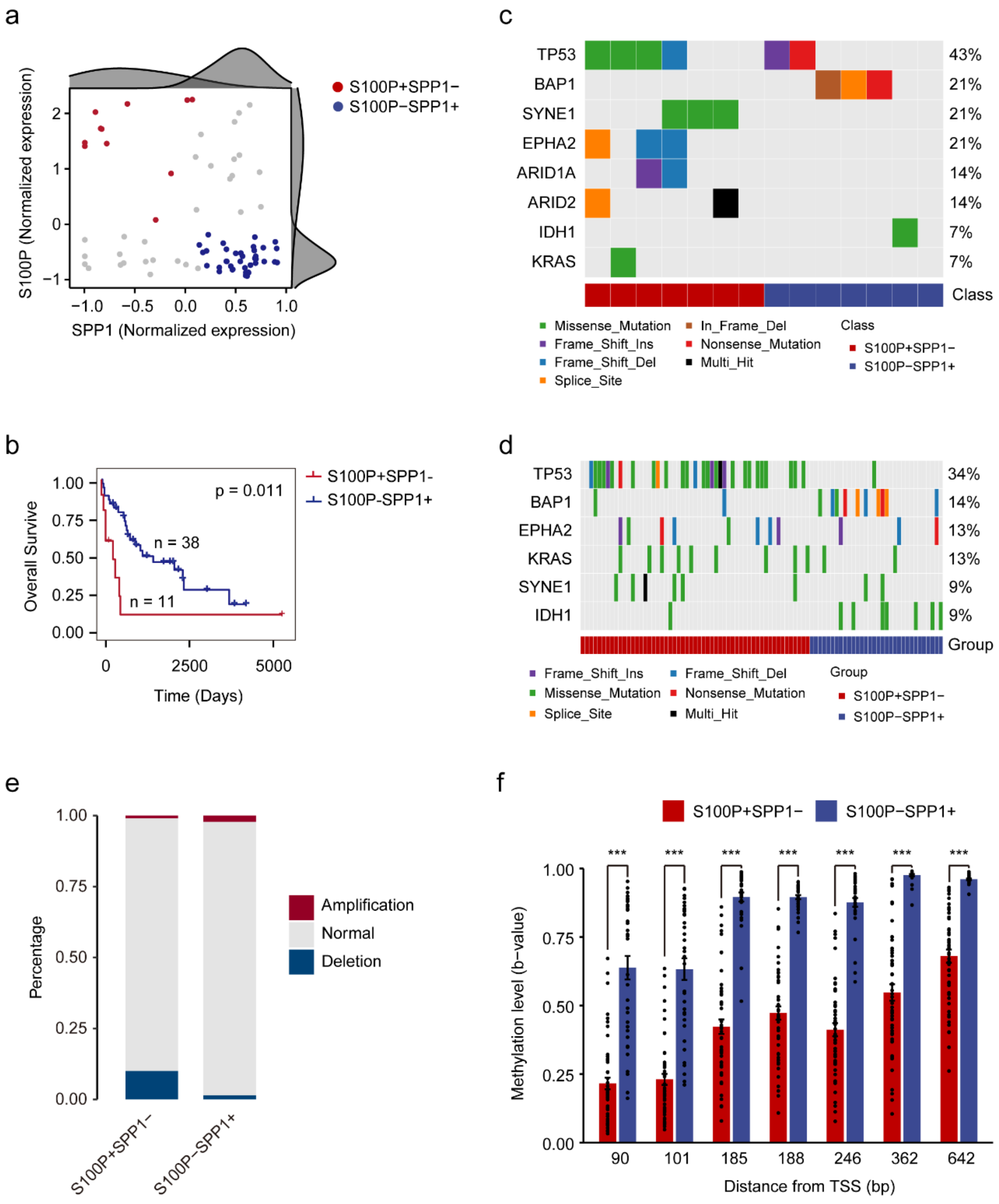

Supplementary Figure. 5 Genomic and epigenetic differences between S100P+SPP1- and S100P-SPP1+ iCCA. a Scatterplot of S100P and SPP1 expression from Job *et al*.'s cohort (Job S, *et al,* 2020, Hepatology). A Gaussian mixture model with two mixture components was used to identify S100P+/- and SPP1+/- patients (right and top distribution curves). **b** Kaplan-Meier survival

plots of the S100P+SPP1- and S100P-SPP1+ groups based on Job *et al*.'s cohort (Job S*, et al,* 2020, Hepatology). Two sided log-rank test. **c** Oncoplot of selected driver genes in 14 iCCA patients. Different types of genomic alterations were shown by different colors. **d** Oncoplot of genomic alterations of 87 specimens from Jusakul et al. dataset. **e** CNV inferred from scRNA-seq on S100P-located position in S100P+SPP1- and S100P-SPP1+ iCCA. Red and blue colors indicate amplification and deletion, respectively. **f** Methylation levels of probes in S100P promoter region based on Jusakul *et al*. dataset (Jusakul *et al*, 2017, Cancer Discovery). Probes were ordered by their distance to the TSS of S100P. S100P+SPP1-, n = 58 samples; S100P-SPP1+, n = 35 samples. (****P* < 0.001; two-sided Wilcoxon-rank sum test; 90bp: *P* = 6.2e-11; 101bp: *P* = 3.1e-11; 185bp: *P* = 2.9e-14; 188bp: *P* = 6.9e-15; 246bp: *P* = 1.7e-14; 362bp: *P* = 8.5e-15; 642bp: *P* = 5.3e-15). Data represented the mean ± SEM. Source data are provided as a Source Data file.

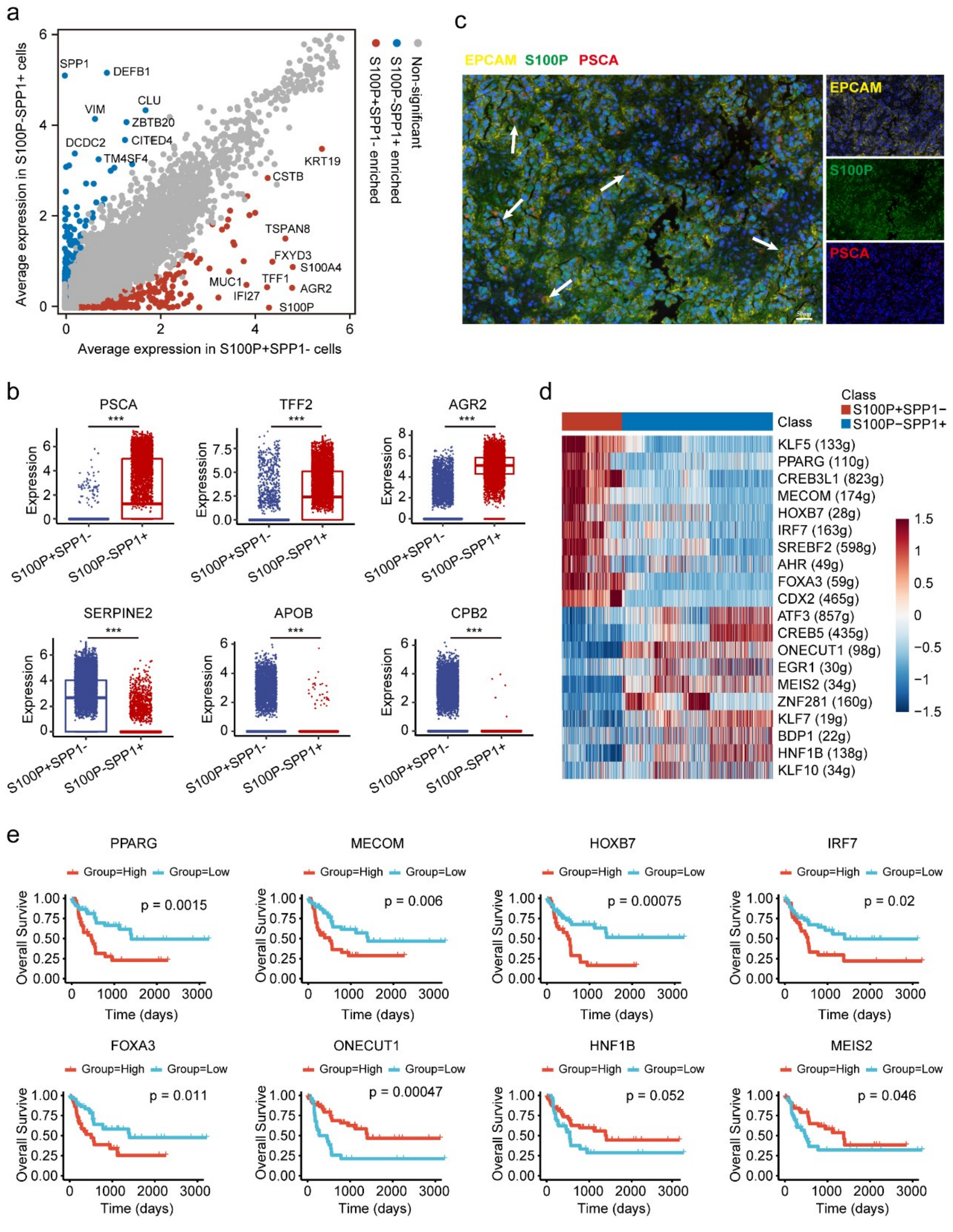

Supplementary Figure. 6 Different gene expression profiles between S100P+SPP1- and S100P-SPP1+ tumor cells. a Scatterplot comparing average expression of genes between the S100P+SPP1- and S100P-SPP1+ tumor cells. Red represents S100P+SPP1- enriched genes while blue

represents S100P-SPP1+ enriched genes. **b** Boxplot showing the differential gene expression between S100P+SPP1- and S100P-SPP1+ tumor cells. Each dot represents a single cell. S100P+SPP1-, n = 5,669 cells; S100P-SPP1+, n = 14,123 cells. (****P* < 0.001; two-sided Wilcoxon-rank sum test; PSCA, TFF2, AGR2, SERPINE2, APOB, and CPB2: *P* < 2.2e-16). The central mark indicates the median, and the bottom and top edges of the box indicate the first and third quartiles, respectively. The top and bottom whiskers extend the boxes to a maximum of 1.5 times the interquartile range. **c** Representative mIHC images to show the distribution of EPCAM+S100P+PSCA+ tumor cells in iCCA: EPCAM (yellow), S100P (green), PSCA (red), and DAPI (blue). White arrows (EPCAM+S100P+PSCA+). Experiment was repeated once with similar results. Scale bar, 50μm. **d** Heatmap showing activity of transcription factors (rows) in S100P+SPP1- and S100P-SPP1+ cells (columns), as estimated by SCENIC. **e** Kaplan-Meier survival plots of iCCAs from Jusakul *et al*.'s dataset (Jusakul *et al*, 2017, Cancer Discovery). Patients were grouped by expression level (median as cutoff) of differentially expressed transcription factors between S100P+SPP1- and S100P-SPP1+ tumor cells. Two-sided log-rank test. Source data are provided as a Source Data file.

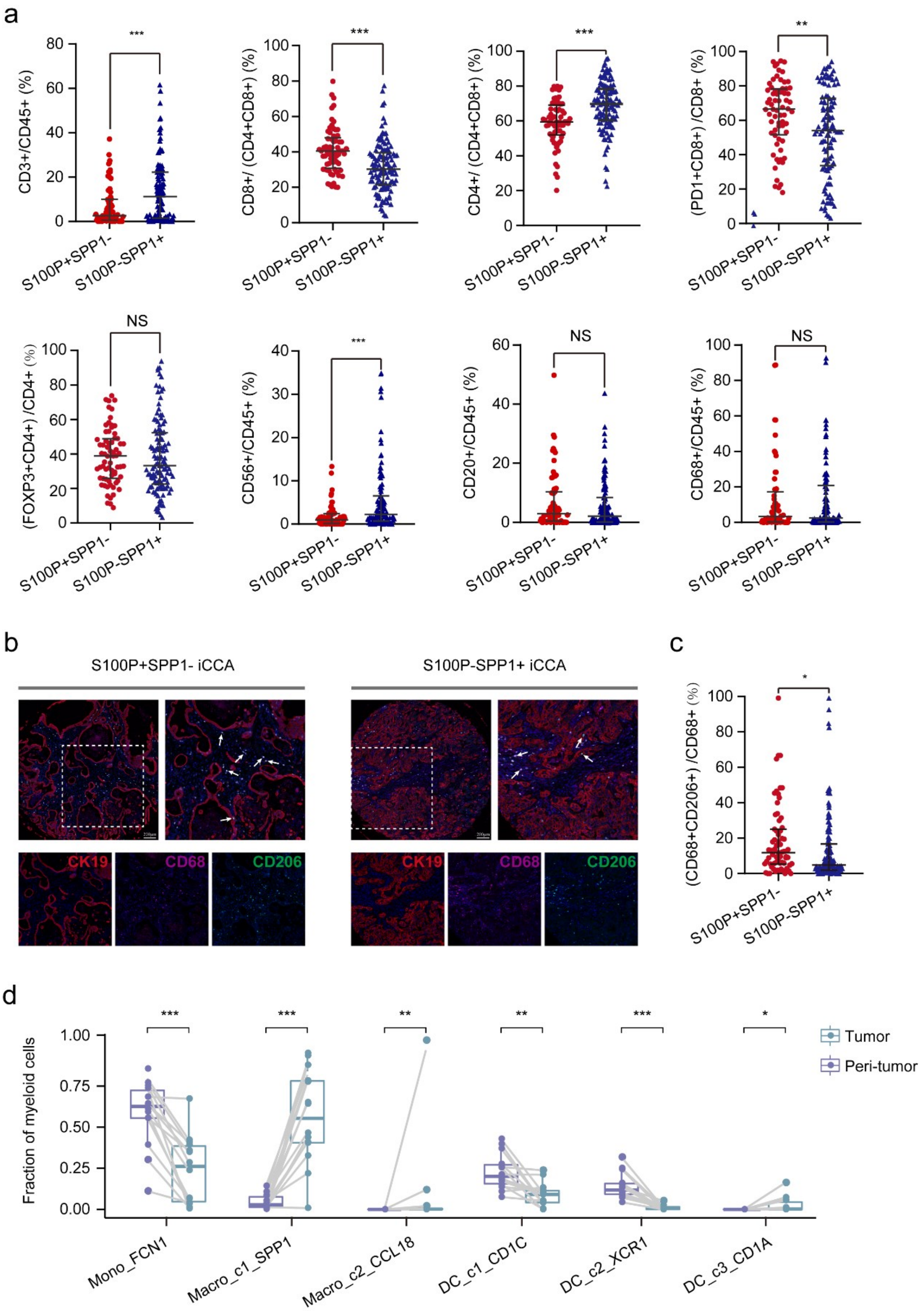

Supplementary Figure. 7 Difference of immune infiltration between S100P+SPP1- and S100P-SPP1+ iCCA. a Comparisons of the proportions of

T cell subsets, CD20$^+$ B cells, CD68$^+$ macrophages and CD56$^+$ NK cells within CD45$^+$ cell between S100P+SPP1- and S100P-SPP1+ iCCA in TMA cohort (S100P+SPP1- n = 68, S100P-SPP1+ n = 118; $^{**}P < 0.01$, $^{***}P < 0.001$; two-sided Mann-Whitney U test; CD3+/CD45+ (%): $P = 0.0007$; CD8+/(CD4+CD8+)(%); $P < 0.0001$; CD4+/(CD4+CD8+)(%); $P < 0.0001$; (PD1+CD8+)+/CD8+(%); $P = 0.0042$; (FOXP3+CD4+)/CD4+(%); $P = 0.5196$; CD56+/CD45(%); $P < 0.0001$; CD20+/CD45(%); $P = 0.3454$; CD68+/CD45(%); $P = 0.8890$; NS: not significant). **b** Representative mIHC images to show the distribution of CD68$^+$CD206$^+$ macrophages in S100P+SPP1- and S100P-SPP1+ iCCA from TMA cohort (n = 201): CK19 (red), CD68 (purple), CD206 (green), and DAPI (blue). White arrows (CD68$^+$CD206$^+$). Experiment was repeated once with similar results. Scale bar, 200μm. **c** Statistical results of CD68$^+$CD206$^+$ within CD68$^+$ macrophages between S100P+SPP1- and S100P-SPP1+ iCCA in TMA cohort (S100P+SPP1- n = 68, S100P-SPP1+ n = 118, $^*P < 0.05$; two-sided Mann-Whitney U test; (CD68+CD206+)/CD68+(%); $P = 0.019$). **d** Boxplot showing the fraction of myeloid subgroups in iCCA peri-tumor and tumor. (Peri-tumor n = 14, Tumor n = 14, $^*P < 0.05$, $^{**}P < 0.01$, $^{***}P < 0.001$, Wilcoxon matched-pairs signed rank test, Mono_FCN1: $P = 0.00012$; Macro_c1_SPP1: $P = 0.00024$; Macro_c2_CCL18: $P = 0.0092$; DC_c1_CD1C: $P = 0.0012$; DC_c2_XCR1: $P = 0.00012$; DC_c3_CD1A: $P = 0.014$). Data are presented as median with interquartile range (**a** and **c**). The central mark indicates the median, and the bottom and top edges of the box indicate the first

and third quartiles, respectively. The top and bottom whiskers extend the boxes to a maximum of 1.5 times the interquartile range. Source data are provided as a Source Data file.

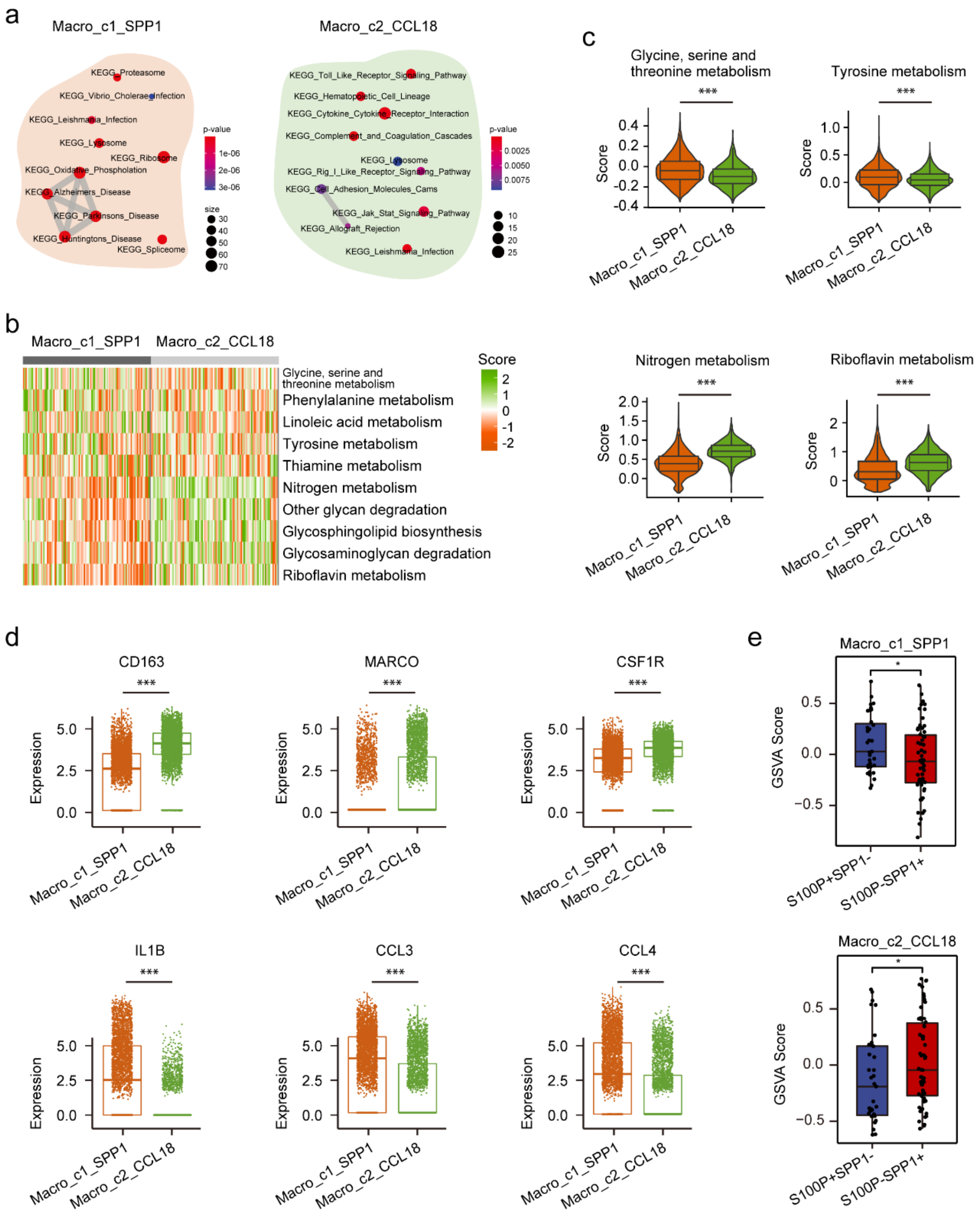

Supplementary Figure. 8 Different subsets of macrophages infiltrated in iCCA^{phl} **and iCCA**^{pps}**. a** Pathway enrichment analysis of highly expressed genes between Macro_c1_SPP1 and Macro_c2_CCL18 macrophages. KEGG gene sets were used to perform the pathway enrichment analysis. **b** The heatmap of metabolic pathway score between Macro_c1_SPP1 and

Macro_c2_CCL18 macrophages. **c** The expression of selected metabolic pathways between Macro_c1_SPP1 and Macro_c2_CCL18 macrophages. Macro_c1_SPP1, n = 4,016 cells; Macro_c2_CCL18, n = 3,447 cells. (***P < 0.001; two-sided Wilcoxon-rank sum test; Glycine, serine and threonine metabolism, Tyrosine metabolism, Nitrogen metabolism, and Riboflavin metabolism: P < 2.2e-16). **d** Boxplot showing the expression of selected genes in Macro_c1_SPP1 and Macro_c2_CCL18. Macro_c1_SPP1, n = 4,016 cells; Macro_c2_CCL18, n = 3,447 cells. (***P < 0.001; two-sided Wilcoxon-rank sum test; CD163, MARCO, CSF1R, IL1B, CCL3, and CCL4: P < 2.2e-16). **e** Boxplot showing the overall expression of Macro_c1_SPP1 program (up) and Macro_c2_CCL18 program (down) estimated by GSVA in S100P+SPP1- and S100P-SPP1+ iCCA from Jusakul *et al*. dataset (Jusakul *et al*, 2017, Cancer Discovery). S100P+SPP1-, n = 58 samples; S100P-SPP1+, n = 35 samples. (*P < 0.05; two-sided Wilcoxon-rank sum test; Macro_c1_SPP1: P = 0.027; Macro_c2_CCL18: P = 0.05). The central mark indicates the median, and the bottom and top edges of the box indicate the first and third quartiles, respectively. The top and bottom whiskers extend the boxes to a maximum of 1.5 times the interquartile range (**c**, **d** and **e**). Source data are provided as a Source Data file.

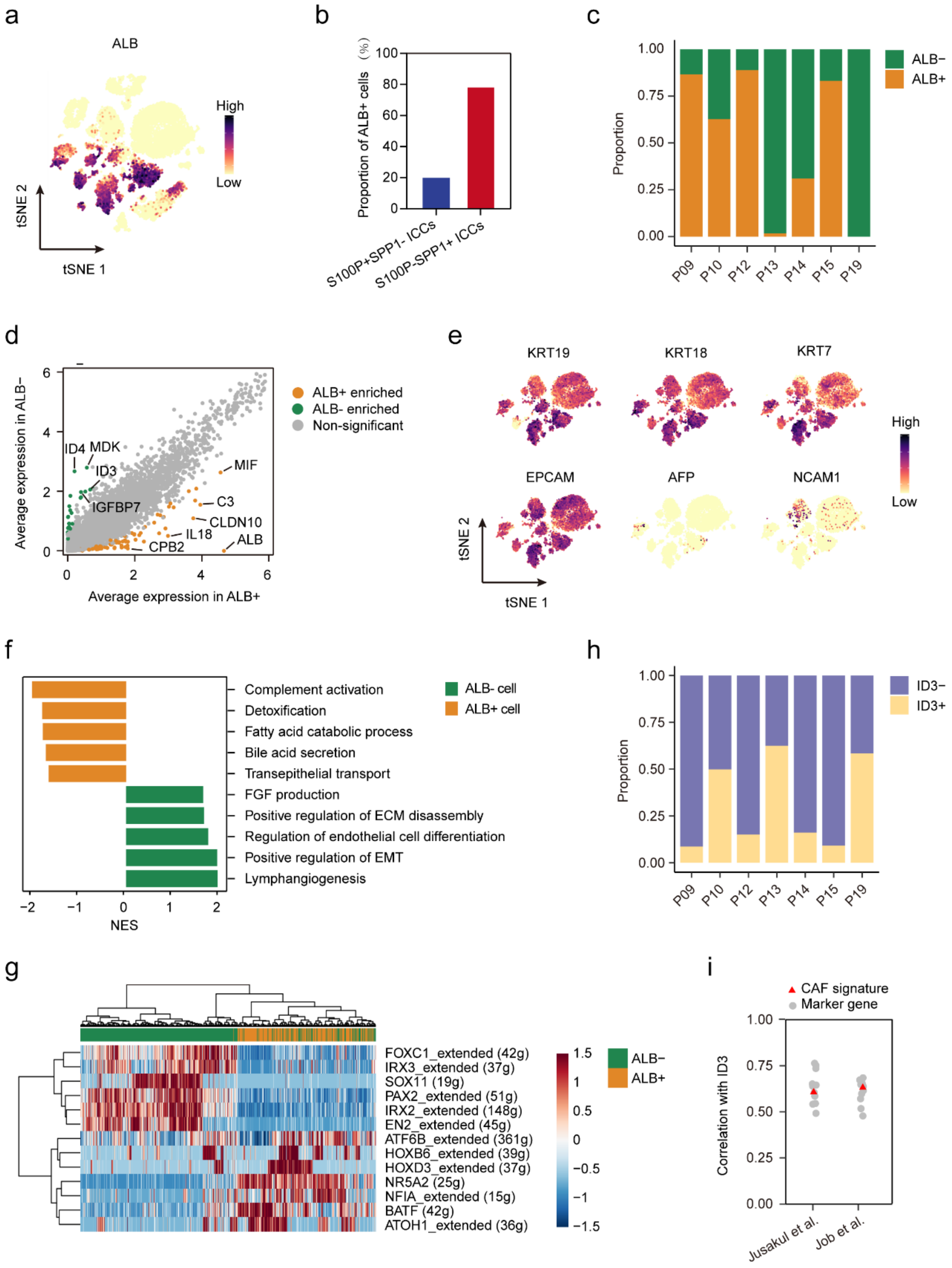

Supplementary Figure. 9 Tumor cells at different stages of differentiation exist in S100P-SPP1+ iCCA. a t-SNE plot showing expression level of ALB in 14 iCCA tumors. **b** Proportion of ALB+ cells in seven S100P+SPP1- iCCAs and seven S100P-SPP1+ iCCAs from scRNA-seq data. **c** Proportion of ALB+ and

ALB- tumor cells in seven S100P-SPP1+ iCCAs. **d** Scatterplot comparing average gene expression level between ALB+ and ALB- tumor cells. Yellow represents ALB+ enriched genes while green represents ALB- enriched genes. **e** t-SNE visualization of selected genes in cells from seven S100P-SPP1+ iCCAs. **f** Top enriched pathways for genes with specific expression in ALB+ and ALB- tumor cells. **g** Heatmap showing activity of transcription factors (rows) in S100P-SPP1+ single cells (columns), as estimated by SCENIC. Shown are the transcription factors with the most significant difference between ALB+ and ALB- tumor cells. **h** Proportion of ID3+ and ID3- tumor cells in seven S100P-SPP1+ iCCAs. **i** Spearman correlation between expression level of ID3 and CAF based on datasets of Jusakul *et al*. (Jusakul *et al*, 2017, Cancer Discovery) and Job *et al*. (Job S*, et al,* 2020, Hepatology). Each grey dot represents a marker gene of CAF as shown in Fig 6B, red triangles represent the overall expression level of CAF signature estimated by MCP-Counter.
