# Supplementary material for: Single-cell transcriptomic analysis suggests two molecularly distinct subtypes of intrahepatic cholangiocarcinoma
Source: Nat Commun. 2022 Mar 28;13:1642. doi: 10.1038/s41467-022-29164-0 (PMC8960779; doi:10.1038/s41467-022-29164-0)
Supplement: Supplementary file 3 — Description of Additional Supplementary Files [file 41467_2022_29164_MOESM3_ESM.pdf]

Description of Supplementary Datasets included in the manuscript “Single-Cell Transcriptomic Analysis Suggests Two Molecularly Subtypes of Intrahepatic Cholangiocarcinoma”.

Supplementary Data 1

The baseline clinical characteristics of our study subjects and overview of the scRNA-seq data used in this study. Relevant to Fig. 1 and Supplementary Fig. 1.

Supplementary Data 2

Gene ranks based on the proportion of positive cells in S100P- cells and S100P+ cells. Relevant to Fig. 2b.

Supplementary Data 3

Clinicopathologic correlation and survival analysis of iCCA patients with S100P+SPP1- and S100P-SPP1+ from TMA cohort. Relevant to Fig. 2e, f.

Supplementary Data 4

Classification of patients in two public datasets based on the expression of S100P and SPP1. Relevant to Fig. 2g-i and Supplementary Fig. 2a, b.

Supplementary Data 5

Differential expression genes between S100P+SPP1- and S100P-SPP1+ tumor cells. Relevant to Supplementary Fig. 6a.

Supplementary Data 6

Marker genes of six clusters of myeloid cells. Relevant to Fig. 6a, b.

Supplementary Data 7

Differential expression genes between SPP1+ALB+ and SPP1+ALB- tumor

cells. Relevant to Supplementary Fig. 9d.

Supplementary Data 8

Anti-human antibodies used in flow cytometry, immunohistochemistry, and Multi-spectral immunohistochemistry. Relevant to Method section.
