# Supplementary material for: Single-cell transcriptomic analysis suggests two molecularly distinct subtypes of intrahepatic cholangiocarcinoma
Source: Nat Commun. 2022 Mar 28;13:1642. doi: 10.1038/s41467-022-29164-0 (PMC8960779; doi:10.1038/s41467-022-29164-0)
Supplement: Supplementary file 12 — Reporting Summary [file 41467_2022_29164_MOESM12_ESM.pdf]

nature portfolio

Corresponding author(s): Qiang Gao

Last updated by author(s): Jan 20, 2022

Reporting Summary

Nature Portfolio wishes to improve the reproducibility of the work that we publish. This form provides structure for consistency and transparency in reporting. For further information on Nature Portfolio policies, see our Editorial Policies and the Editorial Policy Checklist.

Statistics

For all statistical analyses, confirm that the following items are present in the figure legend, table legend, main text, or Methods section.

n/a	Confirmed	
☐	☒	The exact sample size (*n*) for each experimental group/condition, given as a discrete number and unit of measurement
☐	☒	A statement on whether measurements were taken from distinct samples or whether the same sample was measured repeatedly
☐	☒	The statistical test(s) used AND whether they are one- or two-sided<br>*Only common tests should be described solely by name; describe more complex techniques in the Methods section.*
☐	☒	A description of all covariates tested
☐	☒	A description of any assumptions or corrections, such as tests of normality and adjustment for multiple comparisons
☐	☒	A full description of the statistical parameters including central tendency (e.g. means) or other basic estimates (e.g. regression coefficient) AND variation (e.g. standard deviation) or associated estimates of uncertainty (e.g. confidence intervals)
☐	☒	For null hypothesis testing, the test statistic (e.g. *F*, *t*, *r*) with confidence intervals, effect sizes, degrees of freedom and *P* value noted<br>*Give P values as exact values whenever suitable.*
☒	☐	For Bayesian analysis, information on the choice of priors and Markov chain Monte Carlo settings
☒	☐	For hierarchical and complex designs, identification of the appropriate level for tests and full reporting of outcomes
☐	☒	Estimates of effect sizes (e.g. Cohen's *d*, Pearson's *r*), indicating how they were calculated

Our web collection on statistics for biologists contains articles on many of the points above.

Software and code

Policy information about availability of computer code

Data collection	Libraries for ScRNA-seq were generated using the Chromium Single Cell 3' library and Gel Bead & Multiplex Kit from 10x Genomics. Cellanger (v3.1.0) was applied for read mapping and gene expression quantification. Libraries were sequenced on Illumina HiSeq 4000 until sufficient saturation was reached. Whole-exome sequencing was performed on Illunima NovaSeq 6000 after DNA was extracted from iCCA tumor and non-tumor liver tissues from these fourteen patients.
Data analysis	For scRNA-seq, we used the DoubletFinder (v2.0.2), DoubletDetection (Python3), and Scrublet (v0.2.1) algorithms for doublets finding, Seurat (v3) for quality control filtering, data preprocessing, and data visualization, SingleR (v0.1.0) algorithm for cell classifying, QLF model implemented in edgeR (v3.36) for differentially expressed genes identifying, SCENIC (v1.1.0) algorithm for gene regulatory networks profiling, Monocle (v2.8.0) for developmental trajectory analysis. For whole-exome sequencing data analysis, raw sequencing reads were mapped to human genome version 38 (hg38) using BWA-MEM (v0.7.17). After removing duplicated reads, SNV and indel were detected using Mutect2 (v4.1.0) and annotated with Oncotator (1.9.9.0). Copy number alteration (CNA) was identified using FACETS (v0.6.2). InForm software (v2.3, PerkinElmer Inc.) was used for multiplex immunohistochemistry images data analysis.

For manuscripts utilizing custom algorithms or software that are central to the research but not yet described in published literature, software must be made available to editors and reviewers. We strongly encourage code deposition in a community repository (e.g. GitHub). See the Nature Portfolio guidelines for submitting code & software for further information.

Data

Policy information about availability of data

All manuscripts must include a data availability statement. This statement should provide the following information, where applicable:

- Accession codes, unique identifiers, or web links for publicly available datasets
- A description of any restrictions on data availability
- For clinical datasets or third party data, please ensure that the statement adheres to our policy

The raw sequence data reported in this paper (including scRNA-seq and WES data) has been deposited in the Genome Sequence Archive in National Genomics Data Center under the accession number HRA000863, which is accessible at https://ngdc.cncb.ac.cn/gsa-human/browse/HRA000863. The raw sequence data are available for non-commercial purposes under controlled access because of data privacy laws, and access can be obtained by request to the corresponding authors. The request will be passed within one week and then the users will be given a download link valid for 1 year to download the raw data. For public datasets analysis, Jusaka et, al.'s dataset (including 81 iCCAs and 34 ECCs) were retrieved from GSE89749 and GSE89803 (www.ncbi.nlm.nih.gov/geo) and Job et al.'s dataset (including 78 iCCAs) was retrieved from ArrayExpress (accession number E-MTAB-6389, https://www.ebi.ac.uk/arrayexpress/). The remaining data are available within the Article, Supplementary Information or Source Data file. Source data are provided with this paper.

Field-specific reporting

Please select the one below that is the best fit for your research. If you are not sure, read the appropriate sections before making your selection.

[x] Life sciences   [ ] Behavioural & social sciences   [ ] Ecological, evolutionary & environmental sciences

For a reference copy of the document with all sections, see nature.com/documents/nr-reporting-summary-flat.pdf

Life sciences study design

All studies must disclose on these points even when the disclosure is negative.

Sample size	No sample size calculation was performed to pre-determine sample sizes. Sample size for scRNA-seq was determined by the availability of ¡CCA patient samples. We applied scRNA-seq and whole-exome sequencing on tumor and paired adjacent non-tumor liver tissues from fourteen treatment-naïve iCCA patients , yielding a total of 144,878 cells.
Data exclusions	For ScRNA-Seq data, We excluded low quality cells through quality control pipeline, and cell types (e.g. hepatocyte, neutrophil, mast cell and normal epithelial cells) with fewer than 500 cells are also excluded.
Replication	Findings derived from SCRNA-seq analysis were confirmed by Immunohistochemical (IHC) staining in a larger TMA cohort (201 iCCA patients) and two public RNA-seq databases of cholangiocarcinoma (Jusakul A, et al. 2017 Cancer Discov and Job S, et al.2020 Hepatology). For cell experiments (Figure 3e and 3g), at least three replicates were performed and all attempts at replication were successful.
Randomization	Fourteen patients who had liver resection and were pathologically diagnosed as iCCA from January 2019 to January 2020 were randomly selected in this study. These patients were divided into two groups (7 each) based on the expression of S100P or SPP1 (7 were S100P positive and 7 were SPP1 positive). Patients from TMA cohort (201 cases, Figure 2d and 2e) were also divided into S100P+SPP1- or S100P-SPP1+ groups based on the expression of these two genes. For cell experiments (Figure 3), cells were randomly assigned to either treatment or control group.
Blinding	For sRNA-seq data analysis, due to no treatment was done, there was no need for blinding procedure. For cell experiments, blinding was not possible because individual groups of cells received different treatments. IHC staining score was assessed by two independent pathologists who were blinded to group allocation and patients' clinicopathological data during data analysis.

Reporting for specific materials, systems and methods

We require information from authors about some types of materials, experimental systems and methods used in many studies. Here, indicate whether each material, system or method listed is relevant to your study. If you are not sure if a list item applies to your research, read the appropriate section before selecting a response.

Materials & experimental systems

n/a	Involved in the study	
[ ]	[x]	Antibodies
[ ]	[x]	Eukaryotic cell lines
[x]	[ ]	Palaeontology and archaeology
[x]	[ ]	Animals and other organisms
[ ]	[x]	Human research participants
[x]	[ ]	Clinical data
[x]	[ ]	Dual use research of concern

Methods

n/a	Involved in the study	
[x]	[ ]	ChIP-seq
[x]	[ ]	Flow cytometry
[x]	[ ]	MRI-based neuroimaging

Antibodies

Antibodies used	Information of the antibodies used are listed in Supplementary Data 8.<br>1. DRAQ5 (1:200), Cat# 4084, CST;<br>2. DAPI (1:200), Cat# 422801, Biolegend;<br>3. S100P (1:1500 for IHC; 1:3000 for mIHC), Cat# ab133554, Abcam;<br>4. SPP1 (1:2000 for IHC; 1:2000 for mIHC), Cat# ab214050, Abcam;<br>5. Hep-Par1 (1:2000), Cat# ab190706, Abcam;<br>6. MUC5AC (1:1000), Cat# ab3649, Abcam;<br>7. CCL18 (1:1000), Cat# ab104867, Abcam;<br>8. CD68 (1:3000), Cat# 76437, CST;<br>9. CD206 (1:2000), Cat# 91992, CST;<br>10. CREB3L1 (1:1000), Cat# 11235-2-AP, Proteintech;<br>11. CK19 (1:3500), Cat# ab52625, Abcam;<br>12. ID3 (1:2000), Cat# A5375, ABclonal;<br>13. PDGFRβ (1:3000), Cat# ab32570, Abcam;<br>14. ARG1 (1:1000), Cat# ab133543, Abcam;<br>15. EPCAM (1:2000), Cat# ab223582, Abcam;<br>16. PSCA (1:2000), Cat# sc-80654, Santa Cruz Biotechnology;<br>17. CD3 (1:2000), Cat# ab16669, Abcam;<br>18. CD45 (1:2500), Cat# ab40763, Abcam;<br>19. CD20 (1:2500), Cat# ab78237, Abcam;<br>20. CD56 (1:2000), Cat# ab220360, Abcam;<br>21. FOXP3 (1:2000), Cat# ab215206, Abcam;<br>22. CD4 (1:2000), Cat# ab133616, Abcam;<br>23. CD8 (1:2500), Cat# ab237709, Abcam;<br>24. CD68 (1:3000), Cat# ab213363, Abcam;<br>25. PD1 (1:3000), Cat# ab52587, Abcam;
Validation	All antibodies used are commercially available and their manufacturers provided their validation documents. They were validated for flow cytometry, Western blot, IF and/or IHC staining.<br>1. https://www.cellsignal.cn/products/buffers-dyes/draq5/4084?site-searchtype=Products&N=4294956287&Ntt=draq5&fromPage=p|p& _requestid=1465725<br>2. https://www.biolegend.com/en-us/products/dapi-4-6-diamidino-2-phenylindole-dilactate-8108<br>3. https://www.abcam.cn/s100p-antibody-epr6143-ab133554.html<br>4. https://www.abcam.cn/osteopontin-antibody-epr21139-316-ab214050.html<br>5. https://www.abcam.cn/hepatocyte-specific-antigen-antibody-och1e5-ab190706.html<br>6. https://www.abcam.cn/mucin-5ac-antibody-45m1-ab3649.html<br>7. https://www.abcam.cn/ccl18-antibody-ab104867.html<br>8. https://www.cellsignal.cn/products/primary-antibodies/cd68-d4b9c-xp-rabbit-mab/76437?site-search-type-Products&N=4294956287&Ntt=76437&fromPage=plp& requestid=1466111<br>9. https://www.cellsignal.cn/products/primary-antibodies/cd206-mrc1-e219n-rabbit-mab/91992?site-search-type=Products&N=4294956287&Ntt=91992&fromPage=plp<br>10. https://www.ptgcn.com/products/CREB3L1,OASIS-Antibody-11235-2-AP.html<br>11. https://www.abcam.cn/cytokeratin-19-antibody-ep1580y-cytoskeleton-marker-ab52625.html<br>12. https://www.abclonal.com.cn/catalog/A5375<br>13. https://www.abcam.cn/pdgfr-alpha--pdgfr-beta-antibody-y92-c-terminal-ab32570.html<br>14. https://www.abcam.cn/liver-arginase-antibody-epr6672b-ab133543.html<br>15. https://www.abcam.cn/epcam-antibody-epr20532-225-ab223582.html<br>16. https://www.scbt.com/zh/p/psca-antibody-7f5?requestFrom=search<br>17. https://www.abcam.cn/cd3-antibody-sp7-ab16669.html<br>18. https://www.abcam.cn/cd45-antibody-ep322y-ab40763.html<br>19. https://www.abcam.cn/cd20-antibody-ep459y-ab78237.html<br>20. https://www.abcam.cn/ncam1-antibody-epr21827-ab220360.html<br>21. https://www.abcam.cn/foxp3-antibody-epr22102-37-ab215206.html<br>22. https://www.abcam.cn/cd4-antibody-epr6855-ab133616.html<br>23. https://www.abcam.cn/cd8-alpha-antibody-cal66-ab237709.html<br>24. https://www.abcam.cn/cd68-antibody-epr20545-ab213363.html<br>25. https://www.abcam.cn/pd1-antibody-nat105-ab52587.html

Eukaryotic cell lines

Policy information about cell lines

Cell line source(s)	HuCCT1 cell line was purchased from Chinese Academy of Sciences Shanghai Branch Cell Bank (Shanghai, China), RBE cell line was purchased from Cell Resource Center of Tohoku University (Tohoku, Japan), and HEK293 cells were purchased from ATCC.

Authentication	We did not perform cell line authentication.
Mycoplasma contamination	The cell lines tested negative for mycoplasma contamination prior to cryopreservation.
Commonly misidentified lines (See ICLAC register)	None of the used cell lines are placed in the ICLAC register.

Human research participants

Policy information about studies involving human research participants

Population characteristics	All the iCCA patients in this study were Chinese Han nationality. The patient clinicopathological characteristics are presented in Supplementary Data 1.
Recruitment	Fourteen iCCA cases surgically resected from patients were randomly selected with the following criteria: (1) none of the patients received chemotherapy, radiotherapy or any other anti-tumor therapy before surgery; (2) all these cases were pathologically diagnosed as iCCA. Except this, there are no other potential bias for recruitment in our study.
Ethics oversight	This study was conducted in accordance with the ethical standards of the Research Ethics Committee of Zhongshan Hospital with patients' informed consent. (Approval No.: B2017-060R).

Note that full information on the approval of the study protocol must also be provided in the manuscript.
